# miR-27-3p Enhances the Sensitivity of Triple-Negative Breast Cancer Cells to the Antitumor Agent Olaparib by Targeting PSEN-1, the Catalytic Subunit of Γ-Secretase

**DOI:** 10.3389/fonc.2021.694491

**Published:** 2021-06-08

**Authors:** Meng Zhao, Baisheng Sun, Yan Wang, Gengbao Qu, Hua Yang, Pilin Wang

**Affiliations:** ^1^ Department of Breast Surgery, Beijing Tian Tan Hospital, Capital Medical University, Beijing, China; ^2^ Emergency Department, Fifth Medical Center of the General Hospital of the Chinese People’s Liberation Army, Beijing, China; ^3^ Department of Medical Oncology, Affiliated Hospital of Hebei University, Hebei Key Laboratory of Cancer Radiotherapy and Chemotherapy, Baoding City, China

**Keywords:** microRNA-27-3p, Notch pathway, triple-negative breast cancer, olaparib, γ-secretase, PSEN-1

## Abstract

Olaparib has been used in the treatment of triple-negative breast cancer (TNBC) with BRCA mutations. In the present study, we demonstrated the effect of miR-27-3p on the γ-secretase pathway by regulating the sensitivity of TNBC cells to olaparib. miR-27-3p, a microRNA with the potential to target PSEN-1, the catalytic subunit of γ-secretase mediating the second step of the cleavage of the Notch protein, was identified by the online tool miRDB and found to inhibit the expression of PSEN-1 by directly targeting the 3’-untranslated region (3’-UTR) of PSEN-1. The overexpression of miR-27-3p inhibited the activation of the Notch pathway *via* the inhibition of the cleavage of the Notch protein, mediated by γ-secretase, and, in turn, enhanced the sensitivity of TNBC cells to the antitumor agent olaparib. Transfection with PSEN-1 containing mutated targeting sites for miR-27-3p or the expression vector of the Notch protein intracellular domain (NICD) almost completely blocked the effect of miR-27-3p on the Notch pathway or the sensitivity of TNBC cells to olaparib, respectively. Therefore, our results suggest that the miR-27-3p/γ-secretase axis participates in the regulation of TNBC and that the overexpression of miR-27-3p represents a potential approach to enhancing the sensitivity of TNBC to olaparib.

## Introduction

At present, breast cancer (BC) is the most important malignancy threatening female health (1, 2). The main pathological subtypes of BC are endocrine-dependent BC (treated with estrogen receptor-α [ERα] antagonists, including tamoxifen and fulvestrant) and HER2-positive BC (treated with therapeutic antibodies, such as trastuzumab, and small molecules, such as lapatinib) (3–5). The overall prognosis has been significantly improved by the widespread application of effective antitumor drug therapy, but the heterogeneous overall prognosis of triple-negative breast cancer (TNBC) remains unsatisfactory (6, 7). Recently, the PARP inhibitor olaparib was approved for the treatment of TNBC with BRCA mutations (8–10). Although olaparib is considered to have a beneficial effect on patients with TNBC and prolong the survival of patients, there are differences in the sensitivity of individual patients to olaparib, and there are also reports of olaparib resistance (11, 12). Therefore, research and development regarding strategies to achieve more effective olaparib treatment is of great importance.

The Notch pathway not only functions as the key regulator of the cell-fate decision but also induces the resistance of cancerous cells to antitumor strategies, such as radiation therapy and antitumor agents (13, 14). Increasing data have confirmed that the activation of the Notch pathway relates to the occurrence and progress of human cancers (15, 16). Until now, a total of four types of Notch receptors (Notch protein) has been identified: Notch-1, Notch-2, Notch-3 and Notch-4 (17). Five kinds of Notch’s ligands, DLL1, DLL3, DLL4, Jagged1 and Jagged2 bind to Notch protein to activate Notch pathway (18, 19). The Notch-protein is featured as single trans-transmembrane with an extracellular ligand-binding domain and an intracellular domains (NICD) (20). In cancerous cells, the Notch pathway is activated *via* a two-step cleavage process in the presence of ligand-binding (21). ADAMs (a disintegrin and metalloproteinase), including ADAM17 and ADAM10, mediate the first cleavage of Notch, and γ-secretase mediates the second cleavage of Notch; finally, the notch intracellular domain (NICD) is released and translocated to the nucleus to mediate the transcription of certain genes related to drug resistance (22, 23). These genes often encode cellular pro-survival, anti-apoptotic, and epithelial-mesenchymal transition-related factors (24, 25). The activation of the Notch pathway ultimately induces cancerous cells to resist therapeutic strategies (26, 27). Because there are two ADAMs (ADAM17 and ADAM10) that mediate the first cleavage of Notch, the two proteins show mutual compensation in this process, so the second cleavage of Notch, mediated by γ-secretase alone, is an important target for the inhibition Notch pathway activity.

The γ-secretase is considered as an intramembrane aspartate-lyase with multi-subunits, including the PSEN-1, nicastrin subunit (NCSTN), anterior pharynx-defective subunit (APH-1) and presenilin enhancer subunit (PEN-2) (28, 29). Among these subunits, PSEN-1 functions as the catalytic core/subunit for the γ-secretase (30). Inhibition of γ-secretase’s activation has the important anti-tumor properties by blocking of the Notch pathway’s activation and repressing the expression of PSEN-1 is a promising approach for γ-secretase inhibition.

MicroRNA is a type of small non-coding RNA transcribed by RNA polymerase II (31, 32). It can recognize and bind to the 3’UTR of the target mRNA and degrade the mRNA to achieve gene silencing (33, 34). The use of miRNA to inhibit the expression of tumor-related genes has emerged as an important strategy for antitumor therapy (35). The γ-Secretase is a complex containing multiple protein subunits, including PSEN-1, the catalytic center; Pen-2, the activity regulator subunit; and NCSTN, to stabilize the complex (21). In the present study, miR-27-3p was found to inhibit the activation of γ-secretase. The overexpression of miR-27-3p enhanced the sensitivity of TNBC cells, MDA-MB-436 or HCC1937, to olaparib by targeting the 3’UTR of PESN-1.

## Materials and Methods

### Clinical Specimens and Ethical Approval

The use of clinical specimens was approved by the ethics committee of the Beijing Tian Tan Hospital, Capital Medical University. The 30 clinical TNBC specimens (the BRCA mutation subtype) and paired non-tumor tissues were described in our previous publication (35). The usage of the clinical specimens were with the written consent from patients and all the experiments related to the human-derived materials, including clinical specimens and cell lines, were used in accordance with the Helsinki Declaration with the approval from medical ethic committee of Beijing Tiantan Hospital or the/the Hebei Key Laboratory of the Cancer Radiotherapy and Chemotherapy. The sample size used in the present study was adequately powered to detect a pre-specified effect size (1–β: 0.8; α/2: 0.025; P<0.05). The original hypothesis was that the expression of miR-27-3p was not significantly different in healthy tissues as compared with tumor tissue; the alternative hypothesis was that the expression of the targeting gene was significantly different in the healthy tissue as compared with the tumor tissue.

### Vectors, Cell Lines, and Reagents

The full-length sequences of PSEN-1, PSEN-1 with mutated miR-27-3p binding sites, and pre-miR-27 were obtained *via* chemical synthesis. These sequences were cloned and prepared as lentivirus vectors. The vector containing the NICD sequence was a gift from Prof. and Dr. Yingshi Zhang of Shenyang Pharmaceutical University (36). The TNBC cell lines with BRCA mutations, HCC1937 (Cat. No.: 3111C0001CCC 000352) or MDA-MB-436 (Cat. No.: 3111C0001CCC 000471), were purchased from the National Infrastructure of Cell Resources, Chinese Academy of Medical Sciences/Peking Union Medical College, a Chinese government center for biological sample collection. The cells were cultured in DMEM (Dulbecco’s modification of Eagle’s medium) supplemented with 10% FBS. The antitumor agent, olaparib (Cat. No.: S1060), was purchased from Selleck Corporation, Houston, Texas, US. The formulations of olaparib used in the cell-based experiments or animal experiments were described in our previous publication (35).

### Quantitative Real-Time Polymerase Chain Reaction

The Notch pathway-related factors in TNBC tissues, including the clinical specimens and the subcutaneous tumors, or TNBC cells were quantitatively examined using previously established qPCR methods (10, 37). Ribonucleic acid was extracted from the TNBC cells or clinical tissues and reverse transcribed into cDNA. The mRNA expression of these factors was measured *via* qPCR, and β-actin was chosen as the loading control. The primers used in qPCR (37) were (1) CDH1 (E-cadherin), forward primer 5’-CTCCTGAAAAGAGAGTGGAAGTGT-3’, reverse primer 5’-CCGGATTAATCTCCAGCCAGTT-3’; (2) CDH2 (N-cadherin), forward primer 5’-CCTGGATCGCGAGCAGATA-3’, reverse primer 5’-CCATTCCAAACCTGGTGTAAGAAC-3’; (3) vimentin, forward primer 5’-ACCGCACACAGCAAGGCGAT-3’, reverse primer 5’-CGATTGAGGGCTCCTAGCGGTT-3’; (4) ZEB1, forward primer 5’-GATGACCTGCCAACAGACCA-3’, reverse primer: 5’-CCCCAGGATTTCTTGCCCTT-3’; (5) fibronectin, forward primer 5’-CAGGATCACTTACGGAGAAACAG-3’, reverse primer 5’-GCCAGTGACAGCATACACAGTG-3’; (6) SLUG, forward primer 5’-CTTCCTGGTCAAGAAGCA-3’, reverse primer 5’-GGGAAATAATCACTGTATGTGTG-3’; (7) TWIST, forward primer 5’-GTACATCGACTTCCTCTACCAG-3’, reverse primer 5’-CATCCTCCAGACCGAGAAG-3’; (8) BCL2, forward primer 5’-GATCGTTGCCTTATGCATTTGTTTTG-3’; reverse primer, 5’-CGGATCTTTATTTCATGAGGCACGTTA-3’; (9) BIRC2, forward primer 5’-ACATGCAGCTCGAATGAGAACAT-3’; reverse primer 5’-GATTCCCAACACCTCAAGCCA-3’; (10) BIRC3, forward primer 5’-GTGTTCTAGTTAATCCTGAGCAGCTT-3’; reverse primer 5’-TGGAAACCACTTGGCATGTTGA-3’; (11) BIRC5, forward primer 5’-CAAGGACCACCGCATCTCT-3’, reverse primer 5’-AGCTCCTTGAAGCAGAAGAAACA-3’; (12) NICD, forward primer 5’-CCGACGCACAAGGTGTCTT-3’, reverse primer 5’-GTCGGCGTGTGAGTTGATGA-3’; (13) PSEN-1, forward primer 5’-CCATATTGATCGGCCTGTG-4’, reverse primer 5’-GAAGGGCTGCACGAGATAAT-3’; and (14) β-actin, forward primer 5’-CACCATTGGCAATGAGCGGTTC-3’, reverse primer 5’-AGGTCTTTGCGGATGTCCACGT-3’. The qPCR results were presented as a heat map of mRNA expression and obtained *via* the method of Ma et al. (19).

### Cell Survival Analysis

The MDA-MB-436 and HCC1937 cells were cultured and transfected with the indicated vectors. The cells were harvested, seeded into 96-well plates, and treated with the indicated concentrations of olaparib (3 μmol/L, 1 μmol/L, 0.3 μmol/L, 0.1 μmol/L, 0.03 μmol/L, 0.01 μmol/L, or 0.003 μmol/L) for 48 h. The number of cells was examined *via* MTT (3-(4,5)-dimethylthiahiazo (-z-y1) -3,5-di- phenytetrazoliumromide) assay. The inhibition rates were calculated based on the optical density (OD) of cell samples at 490 nm. The I*C_50_* values were calculated based on the rate of inhibition (31, 38).

### Subcutaneous Tumor Model and Ethical Approval

All animal experiments were approved by the Animal Care and Ethics Committee of the Beijing Tian Tan Hospital, Capital Medical University (n=10 for each group, with animals randomly allocated into two groups) and were performed in accordance with the UK Animals (Scientific Procedures) Act, 1986, and associated guidelines. The HCC-1937 or MDA-MB-231 cells were cultured and transfected with the indicated vectors. The cells were injected into nude mice subcutaneously. Then, the mice received olaparib *via* oral administration in accordance with the methods described by Sun HW et al. (39) and Feng et al. (40–42). After olaparib treatment, the samples were harvested from the mice, and the expression of Notch pathway-related factors in tissues was examined by qPCR. The tumor volume was calculated from following equation: (tumor length × tumor width × tumor width)/2 (41, 42). The tumor weight was measured using a precision balance. The heat-map of qPCR results was produced following the method described by Ma et al. (25).

### The Statistical Analysis

The statistical analysis in the presence work were performed by the Bonferroni’s correction with two-way ANOVA methods using SPSS software (Version No. 8.0; IBM Corporation, Armonk, NY, USA). The *IC_50_* values of olaparib were calculated by using the Origin Software (version 6.1, OriginLab, Northampton, Massachusetts, USA). The P<0.05 was considered as statistically significant between the results from indicated two groups.

## Results

### miR-27-3p Targets the 3’UTR of PSEN-1

**Figure 1:**
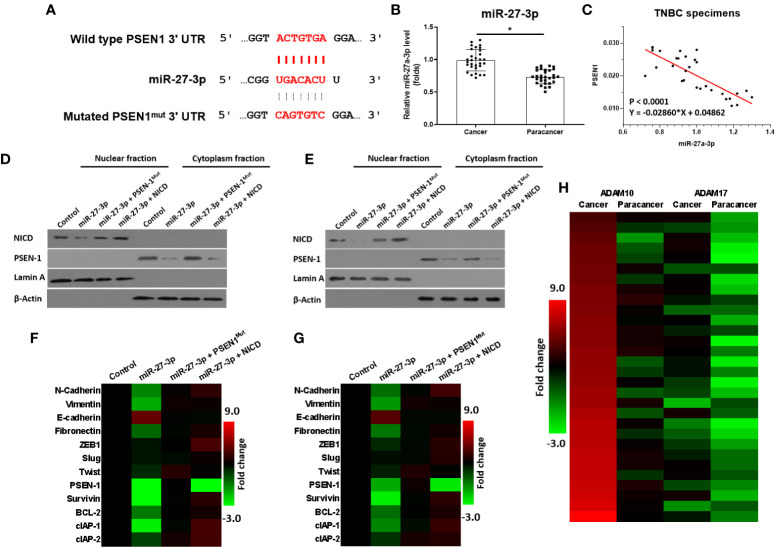
miR-27-3p targets the 3’UTR of PSEN-1. **(A)** miR-27-3p has the potential to target the 3’UTR of PSEN-1. **(B)** The expression of miR-27-3p in TNBC or the paired non-tumor tissues, as determined by qPCR, is presented as a scatterplot. **(C)** The correlation between miR-27-3p and PSEN-1 in TNBC tissues is presented as a scatterplot. **(D, E)** HCC1937 **(D)** or MDA-MB-436 **(E)** TNBC cells were transfected with plasmids, and the subcellular fraction was harvested. The protein expression of PSEN-1 or NICD was examined *via* western blotting. Lamin A was used to indicate the nuclear material, and β-actin was used to indicate the cytoplasm. **(F, G)** HCC1937 **(F)** or MDA-MB-436 **(G)** TNBC cells were transfected with plasmids and harvested for qPCR. The expression of the downstream genes of the Notch pathway are presented as a heat map. **(H)** The expression of ADAM-17 or ADAM-10 in TNBC tissues was examined by qPCR and is presented as a heat map. *P < 0.05.

miR-27-3p was predicted as a microRNA that was potentially able to target the 3’UTR of PSEN-1 and inhibit the activation of the γ-secretase/Notch pathway (**Figure 1A**). As shown in **Figure 1**, miR-27-3p could bind the 3’UTR of PSEN-1. The expression of miR-27-3p was lower in the TNBC specimens than in the paired non-tumor tissues (**Figure 1B**), and the expression of miR-27-3p was negatively correlated with PSEN-1 in TNBC tissues (**Figure 1C**). To confirm the effect of miR-27-3p on PSEN-1, both Western blots of nuclear or cytoplasmic cellular sub-fractions and qPCR assays were performed. As shown in **Figures 1D, E**, the overexpression of miR-27-3p decreased the expression of PSEN-1 but not that of PSEN-1 with a mutated miR-27-3p binding site (PSEN-1^Mut^) in the cytoplasm or the accumulation of NICD in the nucleus of HCC-1937 (**Figure 1D**) and MDA-MB-436 (**Figure 1E**) TNBC cells. The overexpression of NICD almost blocked the effect of miR-27-3p on NICD but not the suppressive effect on PSEN-1 (**Figures 1D, E**). Therefore, miR-27-3p was confirmed to target the 3’UTR of PSEN-1 and thus suppress Notch cleavage.

### miR-27-3p Suppresses the Activation of the Notch Pathway by Targeting the 3’UTR of PSEN-1

The effect of miR-27-3p on the Notch pathway was examined *via* qPCR. As shown in **Figures 1F, G**, the overexpression of miR-27-3p suppressed the expression of pro-survival/anti-apoptosis- or epithelial to mesenchymal transition (EMT)-related downstream genes in the Notch pathway in HCC-1937 (**Figure 1G**) and MDA-MB-436 (**Figure 1H**) cells. Moreover, to further elucidate the roles of the γ-secretase/Notch axis in TNBC, the expression of ADAM17 and ADAM10 was also examined. As shown in **Figure 1H**, the mRNA expression of ADAM17 and ADAM10 was much higher in TNBC as compared with non-tumor tissues (**Figure 1H**). The expression of ADAM10 was higher than ADAM17 in TNBC cells (**Figure 1H**). Therefore, the elucidation of the roles of miR-27-3p and the γ-secretase/Notch pathway in TNBC is of great significance.

### miR-27-3p Enhances the Sensitivity of TNBC Cells to Olaparib by Targeting to the 3’UTR of PSEN-1

**Figure 2:**
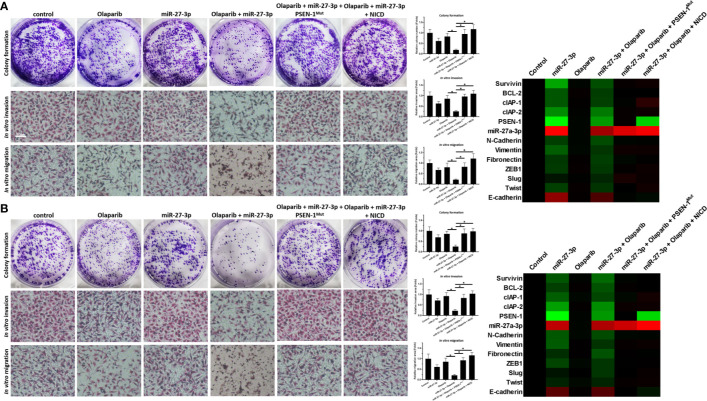
miR-27-3p suppresses the *in vitro* survival or invasion/migration of TNBC cells by targeting the 3’UTR of PSEN-1. **(A, B)** HCC1937 **(A)** or MDA-MB-436 **(B)** TNBC cells were transfected with plasmids or treated with 0.5 μmol/L of olaparib and harvested for colony-formation and Transwell assays. The results are presented as images and quantitative analysis. **(A, B)** The expression of the genes downstream of the Notch pathway in TNBC cells is presented as a heat map. *P < 0.05.

**Figure 3:**
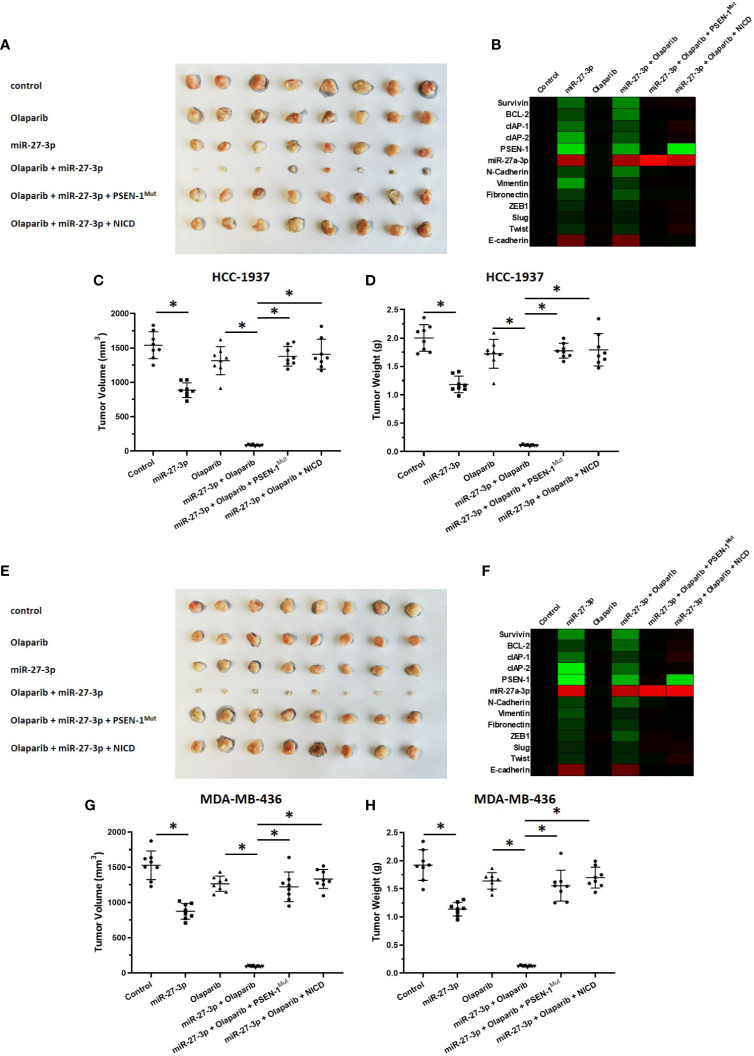
miR-27-3p suppresses the *in vivo* growth of TNBC cells by targeting the 3’UTR of PSEN-1. HCC1937 **(A–D)** or MDA-MB-436 **(E–H)** TNBC cells were transfected with plasmids and injected subcutaneously into nude mice. The mice received a 0.5 mg/kg dose of olaparib *via* oral administration. The results are presented as images or the quantitative analysis of tumor tissues **(A, C–E, G, H)**. **(B, F)** The expression of genes downstream of the Notch pathway in TNBC tissues is presented as a heat map. *P < 0.05.

The ability of miR-27-3p to enhance the sensitivity of TNBC cells to olaparib was examined by multiple assays. As shown in **Figure 2** and **Table 1**, the antitumor effect of olaparib on the survival of TNBC cells and *in vitro* invasion/migration was enhanced in the presence of miR-27-3p. The overexpression of miR-27-3p also inhibited the expression of downstream genes in the Notch pathway (**Figure 2**). Moreover, the overexpression of PSEN-1^Mut^ or NICD almost blocked the effect of miR-27-3p on the survival of TNBC cells, *in vitro* invasion/migration, and downstream genes of the Notch pathway (**Figure 2**). The *in vivo* growth of TNBC was further examined in a subcutaneous tumor model (**Figure 3**). The results showed that TNBC cells formed a tumor in the subcutaneous tissue of nude mice (**Figures 3A–D**). The antitumor effect of olaparib on the subcutaneous growth of TNBC cells was enhanced in the presence of miR-27-3p (**Figures 3A–D**). The overexpression of miR-27-3p also inhibited the expression of downstream genes of the Notch pathway in the tumor tissue (**Figure 3B**). Moreover, the overexpression of PSEN-1^Mut^ or NICD almost blocked the effect of miR-27-3p on the survival of TNBC cells, *in vitro* invasion/migration, and the downstream genes of the Notch pathway in the tumor (**Figure 3B**). Therefore, miR-27-3p enhanced the sensitivity of TNBC cells to olaparib by targeting the 3’UTR of PSEN-1.

**Table 1:** miR-27-3p enhances the sensitivity of TNBC cells to Olaparib.

Groups	HCC-1937	MDA-MB-436
	*IC_50_* values (μmol/L)	
control	0.60 ± 0.20	0.74 ± 0.45
miR-27-3p	0.10 ± 0.06	0.17 ± 0.03
miR-27-3p + PSEN-1^Mut^	0.77 ± 0.28	0.96 ± 0.40
miR-27-3p + NICD	1.05 ± 0.69	0.79 ± 0.08

## Discussion

The first antitumor treatment strategies were mainly cytotoxic chemotherapy drugs, such as paclitaxel or doxorubicin (43, 44). However, recent studies have shown that TNBC is heterogeneous and has multiple pathological subtypes (45). The molecularly targeted drug olaparib (a PARP inhibitor) has been approved for the treatment of BRCA-mutated TNBC (46, 47). Given the broad applications of olaparib in clinical treatment, patients with TNBC have also exhibited resistance to olaparib (48). Therefore, it would be of great significance to reverse the resistance of TNBC to olaparib and increase the sensitivity of TNBC cells to olaparib. The results obtained in this study offer several benefits in terms of treatment. Compared with ADAMs, γ-secretase is a better intervention target for the inhibition of the Notch pathway. This will not only help to expand our understanding of the regulation of Notch in TNBC but also provide more options for TNBC treatment.

The γ-Secretase has three main subunits (21). In this study, transfecting TNBC cells with miR-27-3p, which targets PSEN-1, the catalytic subunit of γ-secretase, inhibited the activity of the Notch pathway and upregulated the sensitivity of cells to the molecularly targeted drug olaparib. Preparing miRNAs as lentiviral vectors to interfere with the expression of specific oncogenes and proto-oncogenes has been confirmed as an effective antitumor treatment strategy (49). The effect of miR-27-3p on PSEN-1 was also examined in target-confirmation studies. The construction of a mutant 3’UTR confirmed the effect of miR-27-3p and the fact that the overexpression of miR-27-3p inhibited the activation of the Notch pathway by targeting PSEN-1. There are four subtypes of Notch protein; these have different extracellular segments (i.e., the N-terminus), but the intracellular segments are highly conserved. Therefore, we prepared nuclear and cytoplasmic sub-fractions and detected the accumulation of the Notch NICD in the nuclear fraction of TNBC cells. Finally, it was confirmed that miR-27-3p regulated Notch protein cleavage, and the influence of miR-27-3p on the Notch pathway was determined by analyzing the EMT, cell pro-survival/anti-apoptosis factors, and other factors downstream of Notch.

Our results mainly focused on the effect of miR-27-3p on Notch pathway in TNBC cells, and miR-27-3p could also regulate some other pathways important for the survival of cancer cells, *e.g.* MMP13, PPARγ, Wnt3a, BTG2 or NOVA1 (50–55). In addition to regulating the survival of malignant tumor cells, miR-27-3p may also modify cancer microenvironment by inhibiting fibroblast viability by targeting NOVA1 (55). Moreover, the effect of miR-27-3p by far not limited to the breast cancer and it has been demonstrated that miR-27-3p may also play an important roles in hepatocellular cancer, gastric cancer or osteosarcoma (54–56). Therefore, our results extended our knowledge about the miR-27-3p. Moreover, the presence work concentrate on the usage of miR-27-3p to inhibit the expression of PSEN-1 to inhibit Notch protein cleavage. In addition, there are other miRs that can inhibit the activity of the Notch pathway through other strategies. For example, miR-3163 can inhibit the expression of ADAM17 by acting on the 3’UTR of ADAM17, and finally inhibit the cleavage of Notch protein in HCC cells (49); miR-34a and others can down-regulate the expression level of Notch-1 protein (26). Some previous publications indicated that miR-27-3p may also interacted with other miRs, e.g. miR-34a-5p (56, 57). These results further confirm the significance of miR-27-3p. In addition to TNBC, Olaparib was also approved for the treatment of ovarian cancer (58, 59). For this reason, the expression levels of miR-27-3p and PSEN-1 in ovarian cancer tissue samples will be further tested in the future, and whether miR-27-3p can upregulate ovarian cancer cells’ sensitivity to Olparib.

## Data Availability Statement

The original contributions presented in the study are included in the article/supplementary material, further inquiries can be directed to the corresponding authors.

## Ethics Statement

The studies involving human participants were reviewed and approved by the ethics committee of the Beijing Tian Tan Hospital, Capital Medical University. The patients/participants provided their written informed consent to participate in this study. The animal study was reviewed and approved by the ethics committee of the Beijing Tian Tan Hospital, Capital Medical University. Written informed consent was obtained from the individual(s) for the publication of any potentially identifiable images or data included in this article.

## Author Contributions 

MZ, HY and PW designed research. MZ, BS, YW and GQ performed the experiments. HY and PW wrote the manuscript with contributions from all authors. All authors contributed to the article and approved the submitted version.

## Conflict of Interest

The authors declare that the research was conducted in the absence of any commercial or financial relationships that could be construed as a potential conflict of interest.
